# One response: Global Outbreak Alert and Response Network supporting the COVID-19 response, Kiribati

**DOI:** 10.5365/wpsar.2024.15.5.1120

**Published:** 2024-06-28

**Authors:** Louise Laurie, Margaret Leong, Toata Titaake Kaufusi, Helen Murdoch, Wendy Snowdon, Sharon Salmon, Peta-Anne Zimmerman

**Affiliations:** a Australasian College of Infection Prevention and Control, Hobart, Tasmania, Australia.; b University Hospital Geelong, Barwon Health, Geelong, Victoria, Australia.; c Suva Regional Office, Pacific Community, Suva, Fiji.; d Ministry of Health and Medical Services, Tarawa, Kiribati.; e World Health Organization Country Liaison Office for Kiribati, Tarawa, Kiribati.; f World Health Organization Regional Office for the Western Pacific, Manila, Philippines.; g Indo-Pacific Centre for Health Security, Department of Foreign Affairs and Trade, Canberra, Australia.; h UNSW Medicine, School of Public Health and Community Medicine, University of New South Wales, Sydney, Australia.; i Griffith University, Gold Coast, Southport, Queensland, Australia.

## Abstract

**Problem:**

In January 2022, Kiribati experienced widespread community transmission of COVID-19, leading to high rates of infection among health-care workers (HCWs), which reduced essential HCWs during a period of increased hospital admissions.

**Context:**

Kiribati, a Pacific island country made up of a remote group of 33 low-lying atolls in the Pacific Ocean, experienced its first surge of COVID-19 cases beginning on 24 January 2022.

**Action:**

Reports of increasing numbers of COVID-19 cases in South Tarawa prompted the Kiribati Ministry of Health and Medical Services to request assistance from the international community, including the World Health Organization’s Global Outbreak Alert and Response Network (GOARN), to support national COVID-19 response operations. Specialists in infection prevention and control (IPC) were deployed to Kiribati in February 2022 to assist the Ministry’s National COVID-19 Taskforce in collaboration with national partners. These specialists helped review and strengthen IPC capacities to accommodate a potential patient surge and consequent demands for medical consumables in health-care facilities in South Tarawa.

**Outcome:**

Strengthened knowledge about and processes for IPC among HCWs prevented health care-associated infections and reduced community disease transmission during the first surge of COVID-19 cases in Kiribati.

**Discussion:**

GOARN has the capacity and ability to rapidly deploy experts to support requests for assistance. Outbreak response activities can be enhanced and sustained by using GOARN’s resources and collaborating with all partners, as necessary.

## PROBLEM

After almost 2 years of restrictions on international travel, introduced at the start of the COVID-19 pandemic, Kiribati recorded its first community-acquired cases of COVID-19 in January 2022. Within a few weeks, South Tarawa, the most populated island, had widespread community transmission of severe acute respiratory syndrome coronavirus 2 (SARS-CoV-2), the virus that causes COVID-19, including among health-care workers (HCWs), propelling a nationwide lockdown and a declaration of a state of disaster by the Government of Kiribati. (*1*)

As hospital admissions escalated, the number of essential front-line HCWs was reduced owing to increased absenteeism due to illness, thus straining an already fragile health-care system. The Kiribati Ministry of Health and Medical Services (MHMS) initiated two requests for international assistance: the first to the Pacific Community and the second through the World Health Organization (WHO) to the Global Outbreak Alert and Response Network (GOARN) to deploy technical experts in infection prevention and control (IPC) to support the national COVID-19 response.

## CONTEXT

**Fig. 1:**
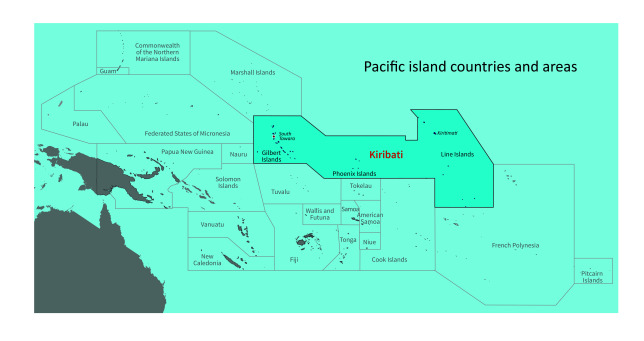
Kiribati shown relative to all Pacific island countries and areas

**Fig. 2:**
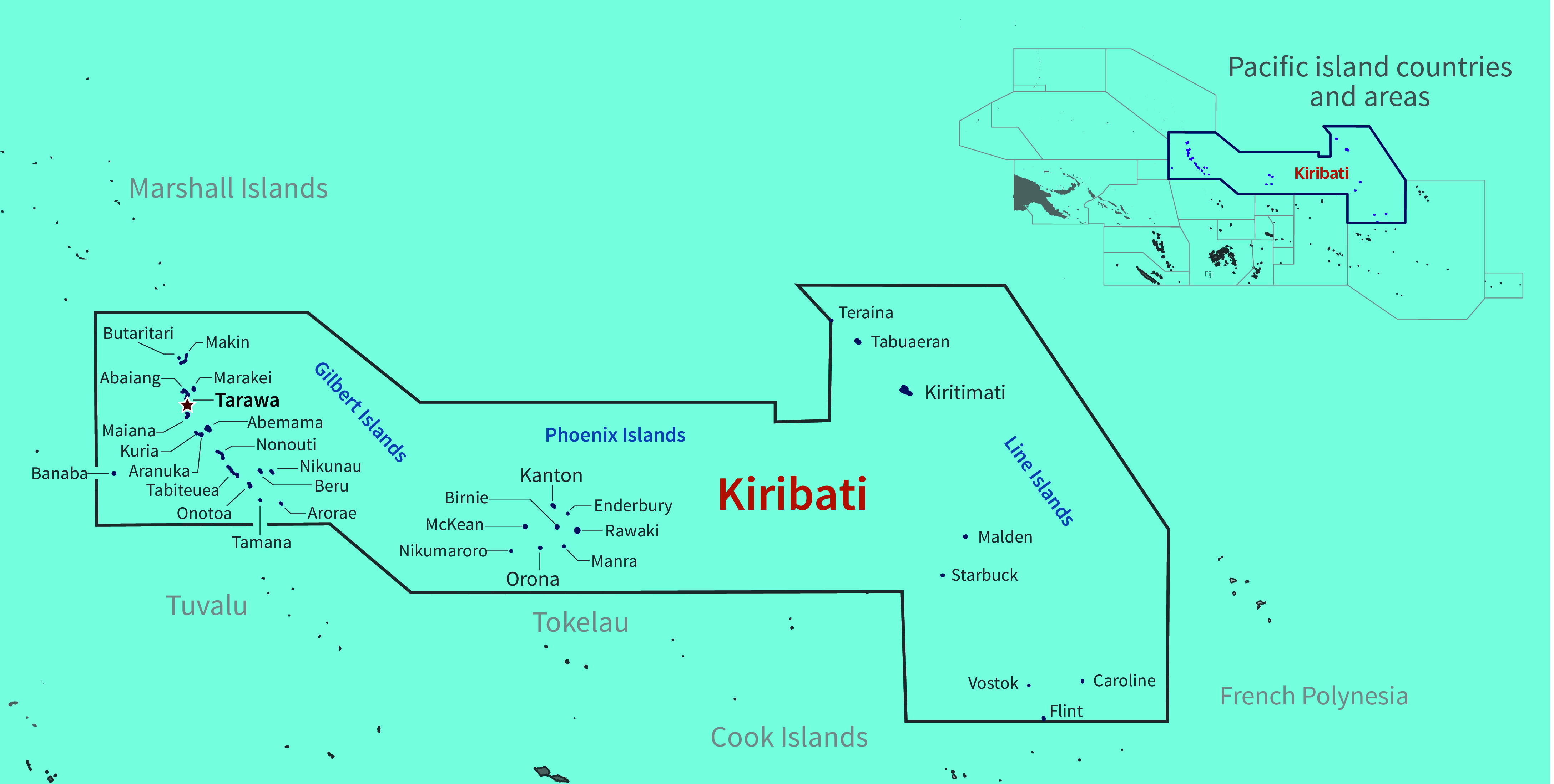
The islands of Kiribati

Kiribati is one of the most geographically isolated countries in the world. Spanning 3.5 million square kilometres of ocean (**Fig. 1**), Kiribati is divided into three groups of islands: the Gilbert Islands, the Phoenix Islands and the Line Islands. Comprising 33 atolls, of which 22 are inhabited (**Fig. 2**), Kiribati is internationally recognized as one of the countries most vulnerable to the impacts of climate change, with most of its land mass less than 2 m above sea level. As of the 2015 census, Kiribati had a population of approximately 110 136, with roughly 50% of the population living on the main island, South Tarawa. (*2*)

[**insert** Figure 1]

[**insert** Figure 2]

**Fig. 3:**
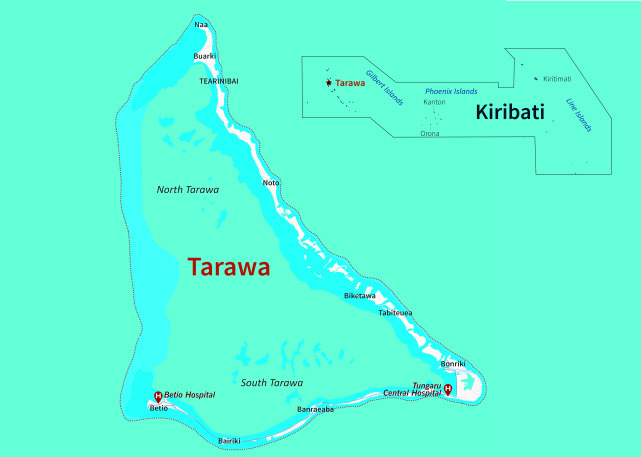
Tarawa and the locations of its two main hospitals in South Tarawa, Kiribati

The Kiribati MHMS provides free health services through a network of health facilities comprising  four hospitals, 30 health centres staffed by medical assistants and 75 clinics staffed by public health nurses. (*2*) Two of the four primary hospitals are located in South Tarawa (**Fig. 3**). Tungaru Central Hospital is a 120-bed referral hospital that provides most of the acute and chronic care in Kiribati, including emergency care, general medical and surgical care, obstetrics and gynaecology, paediatrics and mental health services, as well as additional auxiliary services, including laboratory, medical imaging, pharmacy and physiotherapy services. (*3*, *4*) Betio Hospital serves the populations of Bairiki, Nanikai and Teaoraereke, as well as Betio, providing services such as emergency care, general medicine, maternity care, a pharmacy and dental care.

[**insert** Figure 3]

In May 2021, Kiribati received 24 000 doses of the Oxford–AstraZeneca COVID-19 vaccine. (*5*) These doses supported the initial phase of the vaccination programme that targeted high-risk populations, including HCWs, border control staff, essential workers, individuals with underlying comorbidities and older adults (those aged ≥ 60 years). By January 2022, Kiribati had received additional deliveries of the Oxford–AstraZeneca and Sinopharm vaccines. Eligibility for vaccination was expanded to include all individuals aged ≥ 18 years, with a target population of 71 202 people. As of 4 January 2022, 40 534 people had received two doses of vaccine, and 71 152 people had received at least one dose. (*6*)

In January 2022, 2 years after Kiribati closed its international borders, a chartered flight from Fiji carrying 54 passengers was granted entry into the country. All passengers were tested multiple times, vaccinated and quarantined in Fiji before arriving in Kiribati. (*2*) Upon arrival in South Tarawa, passengers were retested and assessed for COVID-19 symptoms; asymptomatic passengers were quarantined, and symptomatic passengers were isolated. Of the 54 passengers, 36 tested positive for COVID-19 upon arrival and were transported to either a hotel or a designated isolation facility in South Tarawa. Prior to being released from quarantine, all passengers were tested by polymerase chain reaction (PCR) to ensure they were negative for COVID-19. Early in the quarantine period, a small number of COVID-19 cases were detected in the community, and despite efforts to prevent onward transmission, community spread was confirmed soon after.

Evidence of community transmission and reports of infections among HCWs, as well as the increasing pressure on health service delivery, prompted the Kiribati MHMS to request international assistance from GOARN. On 1 February 2022, a request was made for IPC technical support, and on 12 February, GOARN deployed to Kiribati an IPC expert and a multidisciplinary international team via a chartered commercial airline. The emergency response team included two IPC specialists, one from GOARN and one from the Pacific Community; a WHO case management specialist; and two pharmacy logistics specialists from International SOS.

## ACTION

Since the onset of the COVID-19 pandemic, technical experts have been deployed globally through GOARN to support national response operations (**Box 1**). (*7*) In February 2022, IPC specialists from GOARN and the Pacific Community, together with the specialists from International SOS, formed a team to collaborate with the Kiribati MHMS National COVID-19 Taskforce and other national partners to assess, review and strengthen IPC capacities and practices in health-care facilities to reduce SARS-CoV-2 infections associated with such facilities.

Box 1 **About the Global Outbreak Alert and Response Network (GOARN)**

Global Outbreak Alert and Response Network (GOARN)

GOARN is a WHO network of more than 300 technical institutions and networks globally that respond to acute public health events with the deployment of staff and resources to affected countries.
GOARN aims to deliver rapid and effective technical support to prevent and control infectious disease outbreaks and public health emergencies when requested.
For additional information, see https://goarn.who.int/.

To understand Kiribati’s context and needs, the IPC specialists first assessed the two main hospitals in South Tarawa, Tungaru Central Hospital and Betio Hospital, using the Pan American Health Organization’s guidance for assessing IPC practices for COVID-19 isolation areas in health-care facilities. (*8*) Based on the assessment, weekly reports detailing the recommendations for and actions taken to implement IPC were provided to the National COVID-19 Taskforce.

Findings from the assessment revealed problems with segregating infectious patients, the improper use of and limited access to personal protective equipment (PPE), inadequate hand hygiene supplies, and limited environmental cleaning, including cleaning of medical equipment between patients.

To strengthen clinical pathways and prevent health care-associated infections, the MHMS IPC focal point, nursing directors and the WHO clinical management expert worked together to establish more effective patient triage systems. This involved designating physically separate treatment areas for patients presenting with infectious and noninfectious conditions; these were implemented at both hospitals and also in community health-care centres and vaccination clinics.

HCWs were trained in standard and transmission-based precautions, including managing infectious waste through segregation, safe handling, disposal and incineration. (*9*-*13*) Training was delivered through on-site, in-person practical demonstrations, and local HCWs assisted with language translation and provided context.

PPE donning and doffing stations were set up in patient-free areas where there was also access to alcohol-based hand sanitizer and facilities for waste disposal. To ensure there was adequate PPE to meet daily needs for HCWs, a stock inventory management system was established at the two hospitals and overseen by the principal nursing officers. Clean PPE and hand hygiene supplies were collected daily in labelled, sealed, wipeable containers from the dedicated storage space by each ward’s principal nursing officer and distributed to on-duty HCWs. Supply requests were submitted weekly to maintain adequate stock at health-care facilities.

The IPC specialists also coordinated the development of standard operating procedures (SOPs) that were endorsed by the Director of Nursing to provide guidance for IPC training and ensure the sustainability of practices. The SOPs included standard and transmission-based precautions, as well as environmental disinfection, the selection and use of PPE, the management of contaminated linen and waste, and hand hygiene.

At the request of the Kiribati Chief for Environmental Health, the IPC specialists conducted a review of practices at the airport and seaports. Staff reported fainting, dehydration, overheating and fears for their safety while wearing coveralls, gloves and face shields for extended periods in high-wind environments such as airport tarmacs and shipping dockyards. The review recommended the development of an SOP to detail when, what and how to wear and remove PPE, including posters for all front-line staff and the community using these services.

## OUTCOMES

HCWs identified inadequate space and overcrowding in health-care facilities as stress-inducing and leading to an excessive number of sick patients, posing challenges for both the facility and the health-care team. In Kiribati, where HCWs and cleaning staff are predominantly women who also have substantial caregiving responsibilities at home, absenteeism rates were high due to concerns about contracting and transmitting SARS-CoV-2 to their families. IPC specialists provided practical training to improve HCWs’ confidence and skills in administering safe clinical care, thereby lowering the incidence of infections among patients and HCWs. The establishment of an inventory management system streamlined access to PPE and hand hygiene supplies for HCWs, which was achieved through strengthening collaboration among the national pharmacy team, nursing leaders and pharmacy logistics specialists from International SOS. The PPE provided was at times unsuitable; for example, coveralls supplied to workers in a very hot climate can raise concerns about occupational health and safety. (*14*)

The SOPs developed for airport and seaport staff increased their confidence in selecting the appropriate level of PPE for their activity and risk of exposure. Hand hygiene training and improving access to alcohol-based hand sanitizer eliminated the need for continuous glove-wearing for some staff. Switching from heavy-duty coveralls to lighter disposable gowns, and from full-face shields to goggles, also helped to reduce overall discomfort, fatigue, dehydration and hyperventilation among staff.

## Discussion

Kiribati’s request for international assistance was met promptly by GOARN and other organizations with response capabilities. Despite international border closures, restrictions on flights, and strict health and visa requirements, MHMS promptly facilitated the entry of international experts into Kiribati to complement its national response capacity.

Several strategies were used to cultivate strong collaboration among stakeholders. Having a clear allocation of roles and responsibilities within the response team ensured that each member comprehended their role in the response effort. Training sessions, although technical, emphasized teamwork to enhance synergy and coordination. An open and inclusive approach to collaboration was embraced, promoting unity and collective success.

Collaborative joint meetings involving the Kiribati IPC Focal Point, international IPC experts and national partners led to the development of tailored recommendations and interventions for the COVID-19 response. Through efforts coordinated at the national level, key IPC processes were established, including in-person training, the creation of SOPs and implementation of a stock inventory management system. These initiatives enhanced the ability of HCWs in Kiribati to deliver safe clinical care to patients and staff amidst the challenges of the COVID-19 pandemic. However, ongoing efforts are necessary to create a sustainable IPC programme with trained IPC focal points capable of delivering relevant advice and training to HCWs, thereby bolstering IPC capacity and readiness in Pacific island countries and areas.
